# Circulating interleukin-8 and tumor necrosis factor-α are associated with hot flashes in healthy postmenopausal women

**DOI:** 10.1371/journal.pone.0184011

**Published:** 2017-08-28

**Authors:** Wan-Yu Huang, I-Lun Hsin, Dar-Ren Chen, Chia-Chu Chang, Chew-Teng Kor, Ting-Yu Chen, Hung-Ming Wu

**Affiliations:** 1 Institute of Basic Medical Sciences, College of Medicine, National Cheng Kung University, Tainan, Taiwan; 2 Inflammation Research & Drug Development Center, Changhua Christian Hospital, Changhua, Taiwan; 3 Comprehensive Breast Cancer Center, Changhua Christian Hospital, Changhua, Taiwan; 4 Department of Nephrology, Changhua Christian Hospital, Changhua, Taiwan; 5 Division of Statistics, Internal Medicine Research Center, Changhua Christian Hospital, Changhua, Taiwan; 6 Department of Neurology, Changhua Christian Hospital, Changhua, Taiwan; 7 Graduate Institute of Acupuncture Science, China Medical University, Taichung, Taiwan; Katholieke Universiteit Leuven Rega Institute for Medical Research, BELGIUM

## Abstract

**Introduction:**

Hot flashes have been postulated to be linked to systemic inflammation. This study aimed to investigate the relationship between hot flashes, pro-inflammatory factors, and leukocytes in healthy, non-obese postmenopausal women.

**Participants and design:**

In this cross-sectional study, a total of 202 women aged 45–60 years were stratified into one of four groups according to their hot-flash status: never experienced hot flashes (Group N), mild hot flashes (Group m), moderate hot flashes (Group M), and severe hot flashes (Group S). Variables measured in this study included clinical parameters, hot flash experience, leukocytes, and fasting plasma levels of nine circulating cytokines/chemokines measured by using multiplex assays. Multiple linear regression analysis was used to evaluate the associations of hot flashes with these pro-inflammatory factors.

**Settings:**

The study was performed in a hospital medical center.

**Results:**

The mean values of leukocyte number were not different between these four groups. The hot flash status had a positive tendency toward increased levels of circulating IL-6 (*P*-trend = 0.049), IL-8 (*P*-trend < 0.001), TNF-α (*P*-trend = 0.008), and MIP1β (*P*-trend = 0.04). Multivariate linear regression analysis revealed that hot-flash severity was significantly associated with IL-8 (*P*-trend < 0.001) and TNFα (*P*-trend = 0.007) among these nine cytokines/chemokines after adjustment for age, menopausal duration, BMI and FSH. Multivariate analysis further revealed that severe hot flashes were strongly associated with a higher IL-8 (% difference, 37.19%; 95% confidence interval, 14.98,63.69; *P* < 0.001) and TNFα (51.27%; 6.64,114.57; *P* < 0.05).

**Conclusion:**

The present study provides evidence that hot flashes are associated with circulating IL-8 and TNF-α in healthy postmenopausal women. It suggests that hot flashes might be related to low-grade systemic inflammation.

## Introduction

Hot flashes, one of the main bothersome symptoms during menopause [1,2], may occur during the day or night (also known as night sweats). This symptom presents as episodic sensations of heat in the regions of the face and chest, often followed by sweats, and frequently accompanied by palpitation, irritability, and anxiety [2,3]. Hot flashes are shown as a negative factor for quality of life during the climacteric period. The Study of Women’s Health Across the Nation (SWAN) and other studies found that daily and nighty hot flashes were strongly associated with reduced health-related quality-of-life outcomes, including sleep disturbance, anxiety, depression, and reduced cognitive function [4,5].

Recently, hot flashes have been demonstrated to have an impact on physical health outcomes. A number of studies have shown that women who reported hot flashes had impaired lipid profiles [6], endothelial dysfunction [7], increased arthrosclerosis [8,9] and insulin resistance [10,11] during the menopause period. Emerging evidence suggests that hot flashes may be closely linked to the development of metabolic and cardiovascular disease [12]. However, the biological mechanisms underlying these associations remain unclear.

Accumulated evidence suggests that menopause may trigger increase in pro-inflammatory cytokines (e.g. tumor necrosis factor-α (TNF-α), interleukin 6 (IL-6), and interleukin 1, possibly due to the decline of sex hormones such as estrogen [13–15]. Clinical and experimental studies strongly suggest that the increased levels of pro-inflammatory factors may link menopause and subsequent events, such as development of osteoporosis [15] and atherosclerosis [16]. A few studies have evaluated the influence of hot flashes on inflammation [17–21]. Two studies found that increased levels of circulating pro-inflammatory factors, IL-8 and macrophage inflammatory protein-1β (MIP-1β), were higher in premenopausal and menopausal women with hot flashes than in those without hot flashes [17,18]. Other studies reported that hot flashes seemed not significantly related to the changes of pro-inflammatory factors or high-sensitivity C-reactive protein (hs-CRP) [19–21]. Altogether, the effects of hot flashes on systemic inflammation and pro-inflammatory factors seem controversial and need to be further studied.

Inflammation plays a critical role in cardiovascular disease, and the inflammatory factors (e.g. cytokines and chemokines) that are triggered by inflammation are particularly important in atherosclerotic development [22]. Hot flashes are a potential risk factor of cardiovascular disorders. We hypothesized that hot flashes have a role in cytokine dysregulation in menopausal women. To gain better understanding of the links between hot flashes and cytokine expression, a case-control study was conducted to compare nine cytokines/chemokines using multiplex assays between age-matched groups of healthy postmenopausal women with and without hot flashes.

## Materials and methods

### Participants and study design

In this cross-sectional study, the subjects comprised women aged 45 to 60 years who visited the Changhua Christian Hospital for health management reasons during the period January 2013 to January 2016. The present study included postmenopausal women who had at least 12 consecutive months of amenorrhea not due to surgery or other obvious causes, had never experienced hot flashes during menopause stages, or had experienced hot flashes within the three months prior to study entry. Postmenopause was categorized into early (≤ 5 years) and late (> 5 years) postmenopuasal stages, based on Stages of Reproductive Aging Workshop (STRAW) criteria [23]. In addition, their body-mass index (BMI) was within the range of ≥ 18 kg/m^2^ and less than 30 kg/m^2^. Women were excluded if they were in the premenopausal and perimenopausal stages, received hormone therapy, had undergone hysterectomy or bilateral oophorectomy, had a history of chronic systemic diseases including diabetes, hypertension, hyperlipidemia, or thyroid disease or had a BMI < 18 kg/m^2^ or ≥ 30 kg/m^2^, or smoking status. Written informed consent was obtained from all participants. This study was approved by the Changhua Christian Hospital Institutional Review Board (ID: CCH IRB No. 110305). Subject records and information were anonymized and de-identified prior to data collection and statistical analysis.

### Anthropometric measures

Blood specimens of the participants were obtained in the morning after overnight fasting. After centrifugalization at speed of 2500 rpm for 10 minutes, the plasma was aliquoted and stored at −80°C without thawing until assay. Height and weight of participants were measured in light clothing without shoes. BMI was calculated as weight (kg)/height (m)^2^.

### Hot flashes

In the present study, hot flashes were categorized by the severity that women reported. Mild degree was defined as the flash having been a sensation of heat without sweating; moderate degree as the flash having been a sensation of heat with sweating but did not interfere with daily activities; and severe degree as the flash having been a sensation of heat with sweating that caused cessation of activity or interruption of sleep. All participants were required to complete a Menopause-Specific Quality of Life questionnaire [24] to obtain their information related to menopause, such as hot flashes and night sweats. If women had experience of hot flashes that occurred and persisted within the three-month period before study entry, they were asked to fill out a self-reported hot flash diary to report the frequency and intensity of hot flashes that were characterized as mild, moderate or severe over a two-week period [25]. Participants were then divided into one of four groups: Group N comprised postmenopausal women who had never experienced hot flashes or night sweats; Group m comprised women who had experienced mild hot flashes alone at least four days per week but no night sweats; Group M comprised women who had experienced moderate hot flashes but no night sweats at least four days per week; Group S comprised women who had experienced severe hot flashes and/or night sweats at least once per day during this two-week period.

### Measurements of plasma cytokines and chemokines

The plasma levels of interferon-inducible protein-10 (IP10)(also known as CXCL10), monocyte chemoattractant protein-1 (MCP-1) (also known as CCL2), and macrophage inflammatory protein-1β (MIP-1β) (also known as CCL4) were measured using a Millipore cytokine three-plex panel assay (MILLIPLEX MAP Human Cytokine/Chemokine Magnetic Bead Panel)(Milliplex MAP kits, EMD Millipore, Billerica, MA, USA). The plasma levels of interferon gamma (IFNγ), tumor necrosis factor-α (TNF-α), interleukin-1β(IL-1β), IL-6, IL-8 (also known as CXCL8), and IL-17A were determined using a Millipore cytokine seven-plex panel assay (MILLIPLEX MAP Human Cytokine/Chemokine Magnetic Bead Panel) (Milliplex MAP kits, EMD Millipore, Billerica, MA, USA). All analyses were performed by T.Y. Chen according to the manufacturer’s protocol. The data were read using a Luminex 200 system (Luminex, Austin, TX, USA). Values of these cytokines and chemokines were reported as pg/ml. Data on cytokines and chemokines were collected and analyzed using an instrument equipped with MILLIPLEX Analyst software (EMD Millipore). For these nine cytokines/chemokines, the intra-assay laboratory coefficients of variation were less than 8% and the inter-assay coefficients of variation were less than 10%.

### Measurements of sexual hormones and other measures

The concentrations of both estradiol and follicle-stimulating hormone (FSH) were measured using standard procedures at the Department of Laboratory Medicine, Changhua Christian Hospital. Briefly, the concentrations of estradiol and FSH were measured in plasma specimens using the Access Estradiol assay and the Access hFSH assay, respectively, on the Beckman Access Immunoassay system (Beckman Coulter, Fullerton, CA, USA). Values for estradiol were reported as pg/ml, and those for FSH were reported as mIU/mL. The interassay and intraassay laboratory coefficients of variation (CVs) for estradiol were < 8% and 8.3%, respectively, and for FSH were < 8% and 6.2%, respectively. In addition, total WBC number was counted by an automatic Lab instrument at the Department of Laboratory Medicine, Changhua Christian Hospital.

### Statistical analysis

Results are presented as median (IQR: interquartile range). Variables were tested for normal distribution using the Kolmogorov-Smirnov test. One-way analysis of variance (ANOVA) test after logarithmic transformation or Kruskal–Wallis test was used to determine differences between Group N, Group m, Group M, and Group S. Tukey’s *post hoc* tests were then performed to find significant differences between groups. Correlations were determined using Pearson’s correlation analysis. The association between each variable and hot flash status was determined by univariate or multivariate linear regression analysis. The percentage difference in each variable was calculated using the formula (100*(exp(β)-1)) and 95% CI for interpreting coefficients in the multivariate linear regression model. Power analysis was calculated to identify the difference between the three groups (total sample size n = 202) at type one error 0.05 using G*Power software 3.1. Statistical analyses were performed with SPSS software version 19.0.0 (IBM Corporation, Somers, NY, USA). Two-tailed *P* < 0.05 was considered to be statistically significant.

## Results

A total of 202 women fulfilled the entry criteria and were enrolled in this study. Enrolled women were divided into one of four groups based on severity of hot flashes. Participants comprised 39 early and 16 late postmenopausal women in Group N (n = 55), 39 early and 14 late postmenopausal women in Group m (n = 53), 28 early and 12 late postmenopausal women in Group M (n = 40), and 38 early and 16 late postmenopausal women in Group S (n = 54), respectively. There were no significant differences between the groups in median age, number of postmenopausal years since last menstrual period, BMI, total leukocyte number or FSH levels (Table 1).

**Table 1:** Characteristics of the participants by hot flash profiles.

Parameters	Hot flash status				*P*-value	*P*-trend
	None	Mild	Moderate	Severe		
n	55	53	40	54	─	─
Age^†^, years	54 (53,56)	53 (51,56)	52.5 (50,57)	53 (51,55)	0.121	0.093
MP_duration^†^, years	3 (1,6)	2 (1,5)	3 (1,5)	2.5 (1,6)	0.651	0.638
BMI^†^, kg/m^2^	22.9 (21.1,25.3)	23.2 (21.4,25.1)	23.1 (21.25,25.6)	23.35 (21.6,25.4)	0.793	0.489
FSH^†^, mIU/mL	65.2 (45.1,80.3)	56 (33.2,70.8)	58.75 (31.85,72.55)	64.4 (39.9,80.4)	0.140	0.844
Estradiol, pg/mL	< 20	< 20	< 20	< 20	─	─
WBC^‡^, 10^3^/ml	5.4 (4.6,6.2)	5.2 (4.4,6.7)	5.55 (5,6.55)	5.25 (4.5,6.5)	0.561	0.749
IFN-r^‡^, pg/ml	3.76 (1.62,5.33)	2.55 (0.99,5.1)	3.03 (1.83,4.26)	3.6 (1.86,5.52)	0.138	0.271
IL-17A^‡^, pg/ml	1 (0.67,1.64)	0.94 (0.58,1.28)	0.9 (0.62,1.43)	0.94 (0.67,1.77)	0.206	0.215
IL-1β^‡^, pg/ml	0.65 (0.48,0.81)	0.49 (0.39,0.75)	0.59 (0.43,0.77)	0.64 (0.48,0.81)	0.164	0.484
IL-6^‡^, pg/ml	0.21 (0.12,0.32)	0.17 (0.11,0.24)	0.22 (0.15,0.33)	0.25 (0.12,0.41)	0.015	0.049
IL-8^‡^, pg/ml	0.69 (0.57,0.9)	0.65 (0.56,0.95)	0.88 (0.69,1.15)	0.86 (0.64,1.62)*	0.001	<0.001
TNF-α^‡^, pg/ml	0.31 (0.15,0.44)	0.22 (0.15,0.44)	0.38 (0.23,0.55)	0.31 (0.2,0.96)	0.030	0.008
IP-10^‡^, pg/ml	319.98 (273,431)	280.32 (243,368)	340.26 (226,425)	335.68 (252,534)	0.200	0.359
MCP-1^‡^, pg/ml	269.23 (236,307)	264.16 (216,300)	268.08 (226,309)	264.96 (228,315)	0.619	0.521
MIP-1β^‡^, pg/ml	16.32 (11.28,23.33)	15.49 (7.86,22.19)	17.99 (11.15,26.68)	21.93 (8.84,34.89)	0.095	0.040

Data are presented as median (Q1, Q3). Statistical analysis was conducted by Kruskal-Wallis test (marked with †) or ANOVA test (marked with ‡) after logarithmic transformation to compare the mean/median differences between these groups of postmenopausal women with or without hot flashes.

Tukey’s *post hoc* tests were then performed to find significant differences between groups.

*, significant difference between severe hot-flash group and mild hot-flash group (*P* < 0.05), and between severe hot-flash group and non-hot flash group (*P* < 0.05).

Abbreviations: Q, quarter; Q1, 25^th^ percentile; Q3, 75^th^ percentile; MP_duration, menopause period since final menstrual period; FSH, follicle-stimulating hormone; BMI, body mass index; WBC, white blood cell; IFN-γ, interferon gamma; TNF-α, tumor necrosis factor alpha; IL-1β, interleukin one beta; IL-6, interleukin 6; IL-8, interleukin 8; IL-17A, interleukin 17A; IP-10, interferon-inducible protein-10; MCP-1, monocyte chemoattractant protein-1; MIP-1β, macrophage inflammatory protein-1beta.

After logarithmic transformation, one-way ANOVA revealed that there was significant difference in the plasma levels of IL-6 (P = 0.015), IL-8 (P = 0.001), and TNF-α (P = 0.03), and marginal difference in MIP-1β (P = 0.095) among these four groups (Table 1). Simultaneously, the intensity of hot flashes was positively associated with increased levels of circulating IL-6 (P-trend = 0.049), IL-8 (P-trend < 0.001), TNF-α (P-trend = 0.008), and MIP-1β (P-trend = 0.04). Tukey’s *post hoc* tests further revealed that women in Group S displayed significantly higher levels of IL-8 than those in Group m (*P* < 0.05), and N (*P* < 0.05). There were no significant differences in values of these four parameters among groups N, m, and M.

Multivariate linear regression model was used to examine the relationships between hot flashes status and each of the nine cytokines/chemokines after adjusting for follicle-stimulating hormone, body-mass index, age, and menopause duration. Linear regression analysis revealed that hot flash severity had significant effects on IL-8 (*P*-trend < 0.001), and TNF-α (*P*-trend = 0.007) among these nine cytokines/chemokines. Multivariate linear regression analysis further found the differences in IL-8 variable in Group S (% difference (95% confidence interval), 35.48 (13.87,61.18), *P* < 0.001), in Group M (18.5(-1.85,43.07), *P* > 0.05), and in Group m (-1.28(-17.09,17.55), *P* > 0.05), compared to Group N (Table 2), and in TNF-α variable in Group S (35.48 (13.87,61.18), *P* < 0.001), in Group M (18.5(-1.85,43.07), *P* > 0.05), and in Group m (-1.28(-17.09,17.55), *P* > 0.05) compared to Group N (Table 2). However, the association of hot flash status with IL-6 and MIP-1β was not significant. Power analysis revealed that IL-8 had a power of 92.78% and TNF-α had a power of 99.91% in our study. The results indicated that among cytokines/chemokines, IL-8 and TNF-α were highly related to the severity of hot flashes in the postmenopausal women we studied.

**Table 2:** Significant associations of hot flashes with pro-inflammatory factors IL-8 and TNF-α.

Hot flashes severity	IL-8		TNF-α	
	Unadjusted	Adjusted	Unadjusted	Adjusted
None	1	1	1	1
Mild	-1.28 (-17.09,17.55)	0.75 (-15.72,20.44)	-7.97 (-34.79,29.89)	-5.83 (-33.86,34.1)
Moderate	18.5 (-1.85,43.07)	20.98 (-0.18,46.63)	24.47 (-14.19,80.54)	28.89 (-11.91,88.58)
Severe	35.48 (13.87,61.18)^c^	37.19 (14.98,63.69)^c^	49.28 (5.95,110.34)^a^	51.27 (6.64,114.57)^a^
*P*-trend	< 0.001	< 0.001	0.008	0.007

Data are expressed as the percentage difference (95% CI).

Regression coefficients are back-transformed using the formula (100*(exp(β)-1)) to calculate the percentage difference and the 95% CI in cytokine/chemokine index for hot flash status per 1 unit increment.

Linear regression model was adjusted for age, menopause duration, body mass index and follicle-stimulating hormone.

Abbreviations: IL-8, interleukin 8; TNF-α, tumor necrosis factor-alpha.

^a^, *P*-value < 0.05

^c^, *P*-value < 0.001

**Fig 1:**
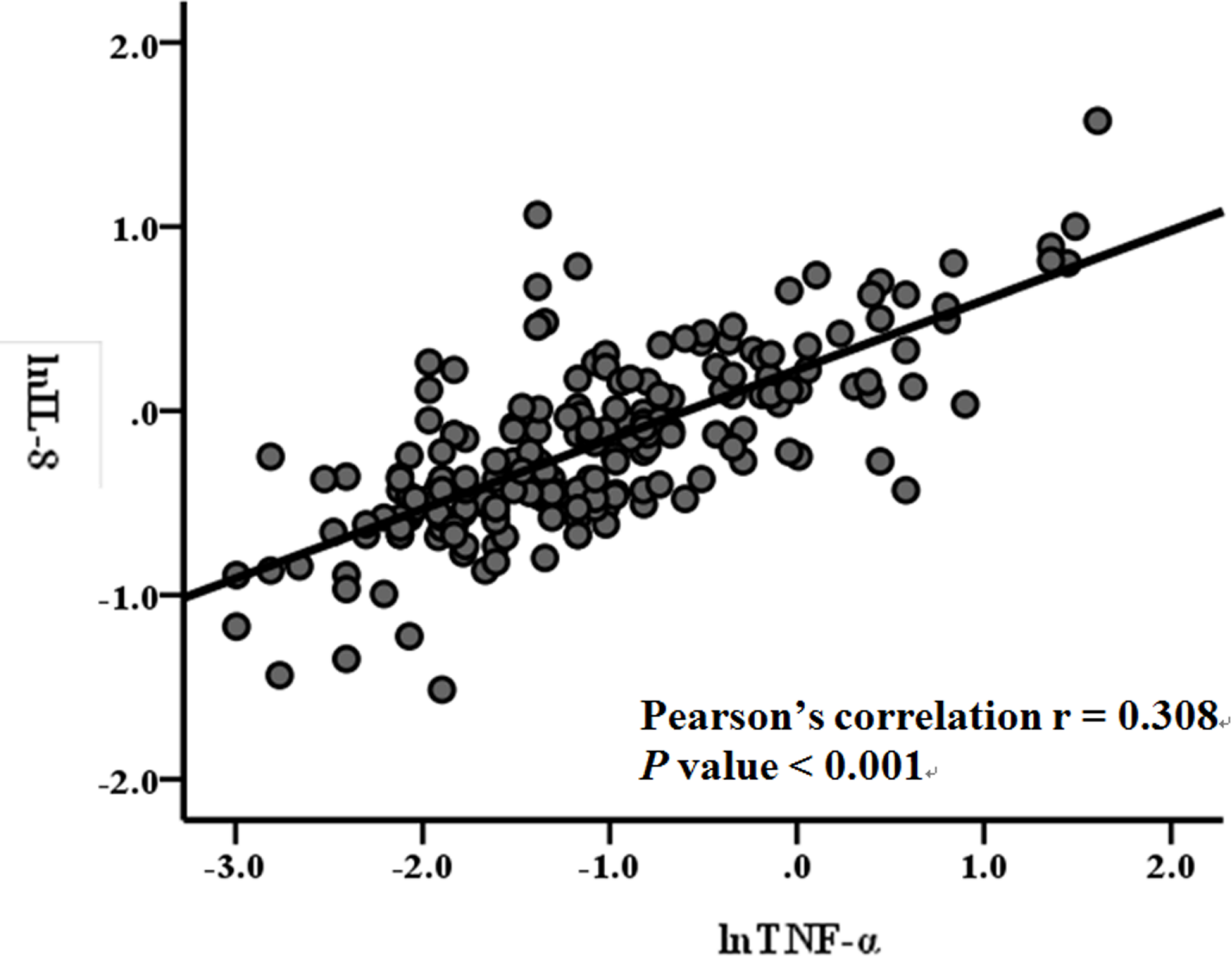
Correlation between IL-8 and TNF-α in the circulating levels. Correlations between IL-8 levels and TN-Fα after log transformation in these 202 postmenopausal women were determined by Pearson’s correlation anal**y**sis.

IL8 gene expression and production in a variety of cell types has been shown to be regulated by TNF-α [26,27]. In the present study, we also examined the relationships of IL-8 with TNF-α. Pearson’s correlation analysis revealed that the correlations of circulating IL-8 levels with TNF-α levels were significant (r = 0.308, *P* < 0.001) after log transformation in these 202 postmenopausal women (Fig 1).

## Discussion

In this investigation of the relationship between hot flashes and inflammation in postmenopausal women, the potential confounding factors were minimized by controlling for the variables that are linked to systemic inflammation (e.g. diabetes [28], estradiol [29,30], hypertension [31], and smoking [32]). We found that hot flash intensity was significantly positively associated with elevated plasma levels of the pro-inflammatory factors IL-8 and TNF-α. These results suggest that hot flashes are linked to low-grade systemic inflammation in postmenopausal women.

The sexual hormone estrogen has been reported to play an important role on production and activity of cytokines including IL-6, IL-1β, TNF-α and other cytokines by binding to nuclear factor-kB or activator protein-1 binding elements in the promoters of these genes in the immune system [13, 29]. On the other hand, estrogen is also a regulator exerting a number of known anti-inflammatory effects, from generating nitric oxide from nitric oxide synthases and regulating immune cells (e.g. lymphocytes) to reducing oxidative stress [30,33]. Estrogen concentration in pre-menopause cyclically changes as well as markedly fluctuates during early and late perimenopause. Therefore, estrogen is a shared risk factor relevant to inflammation from hot flashes. In our postmenopausal participants, estradiol concentrations were undetectably low using our assay method so that our study could minimize the confounding effect of estrogen on the association between hot flash status and the levels of proinflammatory factors in our postmenopausal women.

Recently, Yasui and colleagues found that increased levels of circulating IL-8 and MIP-1β were associated with hot flashes in women at the status of premenopause, menopause transition and postmenopausal stages [17]. Malutan and colleagues measured the pro-inflammatory factor IL-8 and found that serum levels of IL-8 were significantly higher in pre-, peri- and postmenopausal women with severe and moderate hot flashes, compared with those without hot flashes or with mild hot flashes [18]. However, Karaoulanis and colleagues found that serum levels of three cytokines (IL-6, TNF-α, and IL-10) did not significantly change in relation to hot flashes in perimenopausal women with depression [19]. Chedraut and colleagues found that cytokine levels (IL-6 and TNF-α) did not correlate with hot flash scores of the menopause-specific quality of life questionnaire in postmenopausal women with metabolic syndrome [20]. Thurston et al. found no significant association between hot flashes and high-sensitivity C-reactive protein, a systemic inflammation marker, in premenopausal and perimenopausal women [21]. The evidence regarding the role of hot flashes on systemic inflammation seems limited by heterogeneous woman (premenopause, perimenopause, and postmenopause) populations and limited by retrospective or subjective reporting of hot flashes, which may be subject to many biases such as memory bias and other psychological factors. In the present study, we found an association of hot flashes with pro-inflammatory factors IL-8 and TNF-α in the homogeneous postmenopausal population that had undetectably low estradiol levels. Furthermore, our participants provided at least a three-month history of hot flashes prior to study entry and the relatively reliable profiles of hot flashes in frequency and severity. Since a number of characteristics of hot flashes including presence, frequency, duration, and severity may contribute important information about health outcomes in postmenopausal women [12], our study is a more robust investigation of the relationship between hot flashes and systemic inflammation.

IL-8 is a chemokine produced by a wide variety of cells, mainly including innate immune cells (e.g. macrophages), epithelial cells, hepatocytes, and vascular endothelia cells [34]. Circulating IL-8 levels have been reported to be associated with hot flashes in premenopause and menopause women as well as IL-8 receptor levels on neutrophils in late postmenopausal women [35]. The present study detected a consistent association between hot flashes and circulating IL-8 concentrations in our postmenopausal women [17]. Several studies have demonstrated that IL-8 gene expression and production in innate immune cells (e.g. neutrophils and monocytes) and other cell types is regulated by TNF-α, and IL-1β [26,27]. TNF-α is a cytokine that is produced chiefly by activated macrophages and other cell types including lymphocytes, neutrophils, mast cells, Kupffer cells and microglia [36]. In the present study, TNF-α was identified as an additional potential inflammation marker for menopausal hot flashes. Both IL-8 and TNF-α had significant correlation (Fig 1), which might be supported by the biological relation of TNFα-regulation to IL-8 expression [26,27]. We further examined the relationships between IL-8 and its potentially related variables, finding significant association of lnIL-8 with severe hot flashes, and lnTNF-α, but not IL-1β (S1 Table). Based on these findings, our study might suggest that hot flashes have a linkage to increased IL-8 levels potentially in part by mediating activation of TNF-α-induced pathways (S1 Fig).

IL-8 is an important mediator of the immune reaction in neutrophil recruitment and neutrophil degranulation [34], and TNF-α is an important cell signaling protein (cytokine) involved in systemic inflammation. Increasing evidence shows that both factors play key roles as an inflammatory mediator in the development of several systemic chronic diseases including insulin resistance [37], cardiovascular disease [38], and neurodegenerative diseases [39]. Hot flashes have been demonstrated to be a risk factor of systemic diseases such as diabetes, insulin resistance [10,11], and cardiovascular risk [9,12]. Our present study suggests that both IL-8 and TNF-α may be the potential mechanism underlying the link between hot flashes and development of systemic diseases.

Accumulative evidence suggests that menopausal hot flashes are possible to result from an alteration in the CNS thermoregulatory set-point located in the anterior region of the hypothalamus [40,41]. The direction of the thermoregulatory response could depend on the stimulation of specific neurotransmitters (e.g. serotonin and neurokinin B) together with its receptor in brain [2,41,42]. The balance between the serotonin 1A receptor and the serotonin 2A receptor (5-HT2A) might be important for temperature control [2,43]. During menopause, estrogen withdraw could increase sensitivity of the hypothalamic 5-HT2A, leading a change in the thermoregulatory set-point and a hot flush sensation [44]. The role of serotonin signaling on hot flashes is supported by clinical practice of paroxetine, a selective serotonin-reuptake inhibitor (SSRI), for hot flash treatment [45]. In addition, Rance and colleagues have demonstrated that the marked changes in hypothalamic kisspeptin, neurokinin B and dynorphin (KNDy) neurons in postmenopausal women compared with premenopausal women [41,46]. They further proposed that KNDy neurons play a role in the mechanism of flashes. More recently, a phase-2 clinic trial of Neurokinin 3 receptor (NK3R) antagonists support neurokinin B signaling (a hypothalamic neuropeptide) together with its receptor (NK3R) in the aetiology of menopausal hot flashes [47]. Noguchi and colleagues found that increased expression of cytokine-induced neutrophil chemoattractant (CINC), a member of the IL-8 family, was observed in the hypothalamus after injection of luteinizing hormone-releasing hormone agonist in bilaterally ovariectomized rats as a model of hot flashes [48]. Such a result also suggests that CINC might play a role in the homeostasis of body temperature. Our and previous studies found a significant association of hot flashes with systemic inflammation, but are limited to support this relationship of hot flashes and hypothalamic inflammation due to cross-sectional research designs [17–21]. Nevertheless, these new drugs (e.g. SSRI and NK3 receptor blockers) targeting hot flashes may provide a change to clarify whether cytokines (e.g. IL-8) in the hypothalamus play a critical role in the pathophysiology of hot flashes, and whether innate immune system is also a mechanism of action for hot flashes or a combined activated system with hot flashes.

There are several limitations in our study that need to be addressed. First, since this was a cross-sectional study, the investigation was not sufficient to determine whether a causal relationship exists between IL-8, and TNF-α and status of hot flashes. Second, cases with BMI more than 30 kg/m^2^values were excluded from our study. Therefore, the results may not be applicable to other populations (e.g. obese groups). Third, multiplex techniques can measure multiple cytokines in low volume of the same sample simultaneously. However, the concentrations of cytokines or chemokines could be underestimated in this study because of the possible influence of the soluble receptor binding proteins for cytokines.

In summary, we found that hot flashes were significantly associated with elevated levels of circulating IL-8 and TNF-α in healthy postmenopausal women. These results provide evidence showing the linkage of hot flashes to low-grade systemic inflammation. Further longitudinal studies are required to clarify the causal relationships between these inflammatory factors and hot flashes in menopausal women.

## Supporting information

S1 Fig Schematic potential relation between hot flashes, IL-8, TNF-α, and IL-1β. (DOCX)

S1 Table Associations of IL8 with hot flash status, TNF-α, and IL-1β. (DOCX)
