# Supplementary material for: Circulating interleukin-8 and tumor necrosis factor-α are associated with hot flashes in healthy postmenopausal women
Source: PLoS One. 2017 Aug 28;12(8):e0184011. doi: 10.1371/journal.pone.0184011 (PMC5573141; doi:10.1371/journal.pone.0184011)
Supplement: S1 Fig — (DOCX) [file pone.0184011.s001.docx]

**S1 Fig**

***Adj. r* = -0.024**

***Adj. r* = 0.718^c^**

**TNF-α**

**IL-8**

**IL-1β**

**Hot flashes (severe)**

***Adj. r* = 0.152^a^**

***Adj. r* =0.198^a^**

***Adj. r* =0.103**

**S1 Fig Schematic potential relation between hot flashes, IL-8, TNF-α, and IL-1β**

After adjustment for hot flash status, age, menopause duration, body mass index, follicle-stimulating hormone, lnTNF-α, and lnIL-1β, multivariate linear regression analysis revealed significant association between lnIL-8, severe hot flashes, and lnTNF-α (also seen in sTable 1). Based on the evidence of IL-8 gene expression regulated by TNF-α or IL1β, a schematic map shows the potential relation between severe hot flashes, IL-8, TNF-α, and IL-1β.

Abbreviations: IL-8, interleukin-8; TNF-α tumor necrosis factor-alpha; IL-1β, interleukine-1 beta; Adj.r, adjusted standardized coefficient.

^a^, *p*-value < 0.05; ^c^, *p*-value < 0.001
