# Supplementary material for: Circulating interleukin-8 and tumor necrosis factor-α are associated with hot flashes in healthy postmenopausal women
Source: PLoS One. 2017 Aug 28;12(8):e0184011. doi: 10.1371/journal.pone.0184011 (PMC5573141; doi:10.1371/journal.pone.0184011)
Supplement: S1 Table — (DOCX) [file pone.0184011.s002.docx]

**S1 Table Associations of IL8 with hot flash status, TNF-α, and IL-1β**

| Parameters | InIL-8 | |
| --- | --- | --- |
|  | Standardized coefficients | *P*-value |
| Hot flashes severity |  |  |
| Mild | 0.024 | 0.687 |
| Moderate | 0.080 | 0.170 |
| Severe | 0.152 | 0.012 |
| lnTNF-α | 0.718 | < 0.001 |
| lnIL-1β | -0.024 | 0.704 |

The linear regression model was adjusted for hot flash status, age, menopause duration, body mass index, follicle-stimulating hormone, lnTNFα, and lnIL1β.
Abbreviations: IL-8, interleukin-8; TNF-α, tumor necrosis factor-alpha; IL-1β, interleukin-1 beta; ln, natural log.
